# Obstructive sleep apnoea in women with idiopathic intracranial hypertension: a sub-study of the idiopathic intracranial hypertension weight randomised controlled trial (IIH: WT)

**DOI:** 10.1007/s00415-021-10700-9

**Published:** 2021-08-22

**Authors:** Andreas Yiangou, James L. Mitchell, Matthew Nicholls, Yu Jeat Chong, Vivek Vijay, Benjamin R. Wakerley, Gareth G. Lavery, Abd A. Tahrani, Susan P. Mollan, Alexandra J. Sinclair

**Affiliations:** 1 Metabolic Neurology, Institute of Metabolism and Systems Research, College of Medical and Dental Sciences, University of Birmingham, Birmingham, B15 2TT UK; 2 Centre for Endocrinology, Diabetes and Metabolism, Birmingham Health Partners, Birmingham, B15 2TH UK; 3 Department of Neurology, University Hospitals Birmingham NHS Foundation Trust, Birmingham, B15 2TH UK; 4 Birmingham Neuro-Ophthalmology, Ophthalmology Department, University Hospitals Birmingham NHS Foundation Trust, Birmingham, Birmingham, B15 2TH UK; 5 Department of Endocrinology, University Hospitals Birmingham NHS Foundation Trust, Birmingham, B15 2TH UK

**Keywords:** Idiopathic intracranial hypertension, Obstructive sleep apnoea, Screening, Bariatric surgery, Papilloedema

## Abstract

**Objective:**

Obesity is a risk factor for idiopathic intracranial hypertension (IIH) and obstructive sleep apnoea (OSA). We aimed to determine the prevalence of OSA in IIH and evaluate the diagnostic performance of OSA screening tools in IIH. Additionally, we evaluated the relationship between weight loss, OSA and IIH over 12 months.

**Methods:**

A sub-study of a multi-centre, randomised controlled parallel group trial comparing the impact of bariatric surgery vs. community weight management intervention (CWI) on IIH-related outcomes over 12 months (IIH:WT). OSA was assessed using home-based polygraphy (ApneaLink Air, ResMed) at baseline and 12 months. OSA was defined as an apnoea–hypopnoea index (AHI) ≥ 15 or ≥ 5 with excessive daytime sleepiness (Epworth Sleepiness Scale ≥11 ).

**Results:**

Of the 66 women in the IIH: WT trial, 46 were included in the OSA sub-study. OSA prevalence was 47% (*n* = 19). The STOP-BANG had the highest sensitivity (84%) compared to the Epworth Sleepiness Scale (69%) and Berlin (68%) to detect OSA. Bariatric surgery resulted in greater reductions in AHI vs. CWI (median [95%CI] AHI reduction of  – 2.8 [ – 11.9, 0.7], *p* = 0.017). Over 12 months there was a positive association between changes in papilloedema and AHI (*r* = 0.543, *p* = 0.045), despite adjustment for changes in the body mass index (*R*^2^ = 0.522, *p* = 0.017).

**Conclusion:**

OSA is common in IIH and the STOP-BANG questionnaire was the most sensitive screening tool. Bariatric surgery improved OSA in patients with IIH. The improvement in AHI was associated with improvement in papilloedema independent of weight loss. Whether OSA treatment has beneficial impact on papilloedema warrants further evaluation.

**Trial registration number:**

IIH: WT is registered as ISRCTN40152829 and on ClinicalTrials.gov as NCT02124486 (28/04/2014).

**Supplementary Information:**

The online version contains supplementary material available at 10.1007/s00415-021-10700-9.

## Introduction

Idiopathic intracranial hypertension (IIH) is characterised by raised intracranial pressure (ICP) in the absence of an identifiable cause and typically occurs in young women with obesity [1–4]. The incidence of IIH is rising with the increasing prevalence of global obesity [5, 6]. Papilloedema, swelling of the optic nerve, is a diagnostic feature of the condition and can lead to visual loss [7]. Reported symptoms in IIH include headache, transient visual obscurations, pulsatile tinnitus, cognitive disturbances, back and neck pain, and diplopia [1, 8–10]. IIH is further associated with worsening quality of life and increased cardiovascular risk [5, 11].

Co-morbid obstructive sleep apnoea (OSA) has been documented in IIH in cross-sectional studies [12–15]. OSA is characterised by recurrent episodes of complete (apnoea) or partial (hypopnoea) upper airways obstructions resulting in recurrent oxygen desaturation, cyclical changes in heart rate, blood pressure, sympathetic activity and recurrent arousals [16]. Obesity is a risk factor for both OSA and IIH and weight loss can modify both conditions [17–19]. The diagnosis of OSA is important as it is associated with poor quality of life as well as increased morbidity from Type 2 diabetes, cardiovascular disease, hypertension and increased mortality [20]. However, the impact of OSA on IIH clinical course is not clear.

The objectives of this study were as follows: Firstly to determine the prevalence of OSA in a large accurately characterized cohort of active IIH; Secondly to determine the most sensitive screening tool for OSA in IIH; Thirdly to examine effect of weight change on OSA and the impact on IIH clinical outcomes.

## Methods

A sub-study of the IIH: WT trial which was a multi-centre randomised controlled trial comparing the efficacy of bariatric surgery with a community weight management intervention (CWI) in active IIH in the United Kingdom (March 1, 2014 to May 25, 2017) [19, 21].

### Participants

Women (18–55 years old) with active IIH (lumbar puncture opening pressure (LP OP) ≥ 25 cmCSF) diagnosed by the updated modified Dandy criteria with papilloedema [22] and body mass index (BMI) ≥ 35 kg/m^2^ were recruited. Full eligibility criteria and the trial protocol has been previously published [19, 21]. Recruitment bias was avoided by informing and offering recruitment to the sub-study and offering OSA screening to all the IIH: WT participants.

### Assessments

Participants were evaluated at baseline and 12 months. Patient demographics were recorded. Baseline anthropometric measures were performed with body composition scales (Tanita Europe BV, Amsterdam, Netherlands). A headache diary was completed by the participants prior to each visit to quantify the monthly headache days (MHD). Participants underwent optical coherence tomography (OCT) imaging (SPECTRALIS, Heidelberg Engineering, Germany) to evaluate the optic nerve head central thickness (an objective measure of papilloedema) and macular volume ganglion cell layer thickness (a marker of optic nerve axonal loss) [23]. Visual field testing was performed using automated perimetry (Swedish Interactive Threshold Algorithm standard 24–2 strategy, Humphrey Visual Field Analyzer; Carl Zeiss Meditec, Dublin, CA) [23]. Fundus photography (Topcon (Great Britain) Medical Limited, Newbury, United Kingdom) with subsequent papilloedema grading (Frisén grade 0–5) [24] was performed by three blinded neuro-ophthalmology reviewers. ICP was assessed by ultrasound-guided lumbar puncture (LP), with the participant in the left lateral decubitus position, on the same day, after automated perimetry and OCT scanning.

### OSA assessment

At baseline, study participants underwent screening for OSA using the Berlin Questionnaire, [25] Epworth Sleepiness Scale (ESS) [26] and the STOP-BANG questionnaire [27]. All participants were assessed for sleep apnoea at baseline and 12 months using home-based polygraphy which recorded nasal flow, snore, respiratory effort, heart rate, and oxygen saturation (ApneaLink Air, ResMed Corp, CA, USA). The OSA assessments included 2 nights of recordings, the recording which provided the most complete data (≥ 4 h of recording) out of the two nights was used for this analysis. The data were scored by a sleep technician blinded to the participant’s treatment arm and quality controlled by a sleep physician. The sleep studies were scored in accordance with the 2017 updates of the American Academy of Sleep Medicine (AASM) guidelines, [28] which were current at the time of the study (https://aasm.org/clinical-resources/scoring-manual/ Accessed May 2017). Hypopnoeas were defined as a ≥ 3% fall in oxygen saturation from pre-event baseline concomitantly with a ≥ 30% drop in nasal/oral air flow for at least 10 s. Apnoeas were defined as a ≥ 90% drop in nasal/oral airflow for at least 10 s. Central apnoeas were scored in the presence of a ≥ 90% reduction of nasal/oral air flow and cessation of respiratory effort derived from thoracic belt for at least 10 s.

OSA was defined according to the American Academy of Sleep Medicine criteria as an AHI ≥ 15 or AHI ≥ 5 with excessive daytime sleepiness (EDS) defined as ESS ≥ 11 [29]. OSA severity was classified as mild (AHI ≥ 5 and < 15 events/hour), moderate (AHI ≥ 15 and < 30 events/hour) and severe (AHI ≥ 30 events/hour) [29].

### Weight loss interventions

Participants were randomised to either a bariatric surgery pathway or a community weight management intervention (CWI) at baseline (1:1). Randomisation was conducted by a computer-generated randomisation list with allocation to trial arm. The choice of surgical type (laparoscopic adjustable gastric banding, Roux-en-Y gastric bypass or laparoscopic sleeve gastrectomy) in the bariatric surgery pathway arm was made between the surgeon and the participant based on the participant’s health and preference. Participants randomised to the CWI programme were provided with vouchers that exempt them from paying for 52 consecutive and specified weeks of their local WeightWatchers™ group and allowed access to the WeightWatchers™ online and mobile tools for 12 months.

### Statistical analysis

This was a pre-planned, exploratory sub-study of the IIH: WT trial and we report the primary analysis of the data [19, 21]. The sub-study was not powered to observe differences in OSA outcomes after the weight loss intervention. As our data was non-parametric, median and interquartile range (IQR) were used. Wilcoxon signed-rank test was used to compare clinical characteristics of IIH patients between baseline and 12 months. For continuous variables, Mann–Whitney U test was used to compare differences between each study arm (CWI and bariatric surgery) at 12 months. Fisher’s exact test and Kendall’s tau-b were used to compare categorical and ordinal data, respectively. Spearman correlations and multiple regression were used to examine associations and data were log transformed where appropriate. Additional linear regression statistical assumptions were checked and adhered to. Missing data was excluded from analysis in this sub-study as imputation was judged as likely to lead to bias in this exploratory data set.

A two-tailed *p*-value of < 0.05 was considered statistically significant. Statistical analysis was performed using the SPSS statistics 26 package (SPSS Inc., Chicago, IL, USA).

### Standard protocol approvals, registrations, and patient consents

Participants provided written informed consent to participate in the study. The National Research Ethics Committee West Midlands –The Black Country approved IIH: WT (14/WM/0011). The trial protocol, to which these patients were recruited to, has been previously published [21]. The study was conducted according to the Declaration of Helsinki.

## Results

### Baseline characteristics

**Fig. 1:**
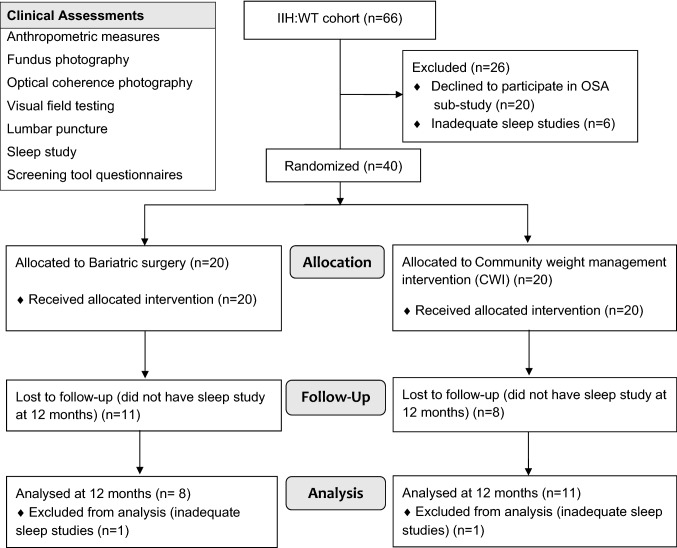
Study flow diagram. *IIH* idiopathic intracranial hypertension, *WT* Weight trial, *OSA* Obstructive sleep apnoea

Of the 66 participants in the IIH: WT, 46 women with active IIH consented to be part of the OSA sub-study. Six IIH patients were excluded from the analysis as their sleep studies were not of adequate quality, hence 40 patients with IIH were included (Fig. 1). The study population consisted of young women with active IIH (Table 1). At baseline, the median (IQR) ICP as measured by LP OP was 34.5 cmCSF (29.3, 39.0). Visual function, papilloedema and headache characteristics are detailed in Table 1.

**Table 1:** Baseline results. Clinical characteristics and sleep study results for IIH patients

Clinical characteristics	IIH, *n* = 40 Median (IQR)
Age (years)	30.2 (27.0, 34.8)
Weight (kg)	112.6 (104.7, 134.3)
Body mass index (kg/m^2^)	43.5 (38.7, 48.0)
Intracranial pressure (cmCSF)	34.5 (29.3, 39.0)
Monthly headache days	28.0 (16.0, 28.0)
OCT global peripapillary retinal nerve fibre layer thickness (µ)	123.0 (98.0, 206.0)
OCT optic nerve head central thickness (µ) (*n* = 27)	617.0 (517.0, 784.0)
OCT Macular volume ganglion cell layer (mm^3^) (*n* = 27)	1.1 (1.1, 1.2)
Visual field perimetric mean deviation (dB)	– 2.3 ( – 3.7, – 1.5)
Frisén grade	2 (1–3)
**Sleep study results (Events/hour, unless otherwise specified)**	
Apnoea-hypopnoea index	7.6 (4.3, 16.0)
Lowest desaturation (SpO2)	87.0 (83.3, 90.0)
Percentage time spent with SpO2 below 90% (%)	0.5 (0, 17.0)
Apnoea index	0.2 (0, 0.6)
Unknown apnoea index	0 (0, 0)
Obstructive apnoea index	0.2 (0, 0.6)
Central apnoea index	0 (0, 0)
Mixed apnoea index	0 (0, 0)
Hypopnoea Index	7.2 (4.0, 15.6)
Oxygen desaturation index	7.7 (3.6, 18.6)

*IIH* idiopathic intracranial hypertension patients, *AHI* apnoea–hypopnoea index (the number of apnoea–hypopnoea events per hour), *SpO2* arterial oxygen saturation (pulse oximetry)

### OSA prevalence in IIH

**Fig. 2:**
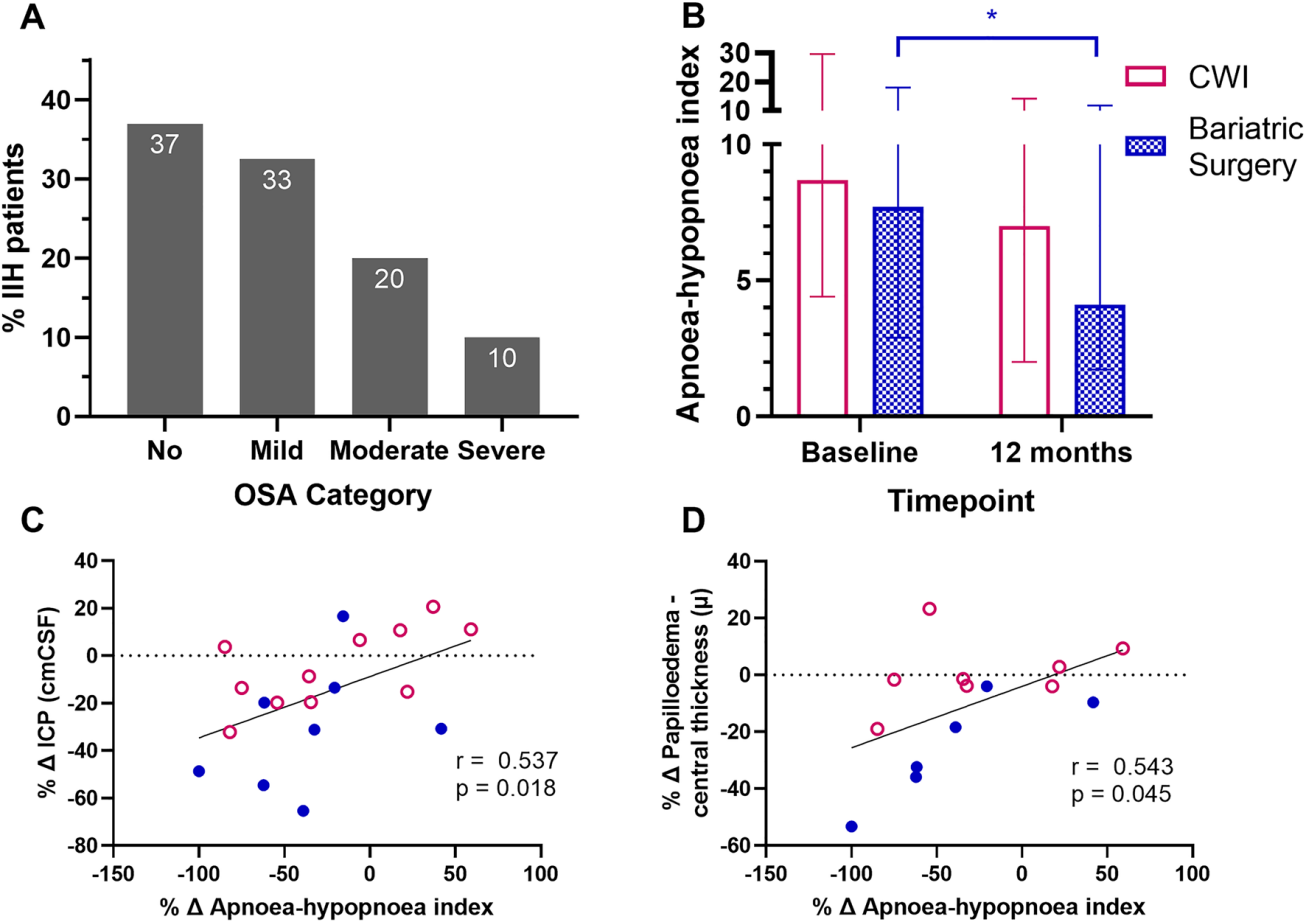
Obstructive sleep apnoea parameters. A. Presence and severity of obstructive sleep apnoea (OSA) based on the apnoea–hypopnoea index in IIH patients. B. Apnoea-hypopnoea index in patients with IIH at baseline comparing the two arms of community weight management intervention (CWI) and bariatric surgery. Level of the bars represents median and error bars interquartile range. Wilcoxon signed ranks test to assess differences between baseline and 12 months in the two groups. Mann–Whitney test to assess differences between arms at 12 months. C, D. Linear regression analysis showing the percentage change (Δ) of apnoea–hypopnoea index against percentage change (Δ) in intracranial pressure and OCT optic nerve head central thickness at 12 months after baseline in IIH patients. Spearman’s rank order used to assess correlations. *Denotes statistical significance *p* < 0.05, difference between apnoea–hypopnoea index of the bariatric surgery group at 12 months compared to baseline. *IIH* idiopathic intracranial hypertension, *ICP* intracranial pressure, *OCT* optical coherence tomography, *AHI* apnoea–hypopnoea index

The AHI, median (IQR) was 7.6 (4.3, 16.0) (Table 1) and AHI ≥ 5 was noted in 25 (63%) IIH patients. 13 (33%), 8 (20%) and 4 (10%) had AHI 5 to < 15; 15 to < 30 and ≥ 30, respectively (Fig. 2A). OSA diagnosis (AHI ≥ 15 or AHI ≥ 5 with EDS, AASM criteria [29]) was made in 19 (47%) of the IIH patients.

### Use of screening questionnaires in IIH

High risk of OSA was identified in 30 (75%) patients using the Berlin questionnaire; 29 (73%) patients using the STOP-BANG questionnaire and 16 (46%) patients using the ESS (*n* = 35) (Table 2). The STOP-BANG questionnaire had the highest sensitivity (84%) to diagnose OSA (AASM criteria) compared to the Berlin 68% and ESS 69% (Table 2). All questionnaires had poor specificity (STOP-BANG 38%, Berlin 19%, ESS 74%). ESS is a component of the AASM criteria to diagnose OSA which includes the presence of excessive daytime sleepiness, therefore a sensitivity analysis for the ESS was conducted using the criterion of an AHI ≥ 15 alone for diagnosis. This revealed a sensitivity 50% and specificity 56% for the ESS.

**Table 2:** Screening tools and test characteristics in IIH patients

Screening tool	IIH patients *n* = 40	Test characteristics	Sensitivity analysis (OSA = AHI ≥ 15)
Berlin			
Low risk	10	Sens = 68%	Sens = 75%
High risk	30	Spec = 19%	Spec = 25%
ESS			
Median (IQR)	9 (5–12)		
Low risk (no EDS)	19*	Sens = 69%	Sens = 50%
High risk (EDS)	16*	Spec = 74%	Spec = 56%
STOP-BANG			
Median (IQR)	4 (2.5–4)		
Low risk	9	Sens = 84%	Sens = 92%
Intermediate risk	2	Spec = 38%	Spec = 29%
High risk	29		

*ESS* epworth sleepiness scale, *EDS* excessive daytime sleepiness, *IIH* idiopathic intracranial hypertension, *AHI* apnoea-hypopnoea index, *Sens* sensitivity, *Spec* specificity

Screening tools for patients with idiopathic intracranial hypertension that undertook a sleep study with the test characteristics for each tool. Sensitivity analysis performed defining OSA as AHI ≥ 15

*IIH patients *n* = 35 for Epworth sleeping scale

### Weight loss intervention

We have previously published results showing significantly reduced weight and ICP amongst the bariatric cohort compared to the CWI cohort at 12 months [19]. In this sub-study we observed analogous results with significantly greater reduction in weight, ICP and papilloedema in the bariatric surgery cohort (Table 3). The AHI significantly improved after 12 months in the bariatric surgery cohort with a median [95% CI] reduction from baseline of  – 2.8 [ – 11.9, 0.7], *p* = 0.017 but not in the CWI cohort (-1.5 [-10.4, 2.2], p = 0.213 (Table 4; Fig. 2B). The difference between the two cohorts at 12 months was not significant, 1.7 [ – 5.7, 7.4], *p* = 0.657, but the study was underpowered for this analysis. We noted a significant correlation at baseline between the AHI and BMI (*r* = 0.360, *p* = 0.022).

**Table 3:** Outcomes in IIH patients

	Baseline	12 months	Difference baseline to 12 months	Difference between arms at 12 months
	Median (IQR), *n*	Median (IQR), *n*	Median [95% CI], *n*; *p*^a^	Median [95% CI] *n*; *p*^b^
Weight (kg)				
All	112.6 (104.7, 134.3), 40	102.7 (85.8, 119.8), 19	– 16.6 [ – 20.6, – 5.6], 19; 0.008	
CWI	113.4 (99.0, 132.1), 20	112.4 (93.2, 115.9), 11	– 0.1 [ – 11.8, 4.0], 11; 0.756	22.6 [ – 14.4, 44.6], 19; 0.014
Bariatric surgery	112.3 (104.8, 135.9), 20	82.9 (69.6–125.1), 8	– 27.2 [ – 34.7, – 16.8], 8; 0.012	
Body mass index (kg/m^2^)				
All	43.5 (38.7, 48.0), 40	39.3 (32.3, 44.9), 19	– 4.9 [ – 7.4, – 2.0,], 19; 0.009	
CWI	43.1 (36.9, 46.9), 20	39.0 (35.5, 46.0), 11	0.1 [ – 1.3, 4], 11; 0.824	7.5 [ – 4.9, 15.2], 19; 0.009
Bariatric surgery	43.7 (39.2, 49.9), 20	30.8 (27.5, 44.6), 8	– 9.3 [ – 12.6, – 5.6], 8; 0.012	
Intracranial pressure (cmCSF)				
All	34.5 (29.3, 39.0), 40	29.0 (26.5, 32.0), 19	– 5.0 [ – 10.5, – 2.1,], 19; 0.009	
CWI	35.5 (29.0, 39.5), 20	30.5 (28.0, 35.0), 11	– 2.5 [ – 6.4, 1.6], 11; 0.266	5.8 [0, 13.5], 19; 0.021
Bariatric surgery	34.3 (30.0, 39.0), 20	26.8 (15.9, 30.5), 8	– 13 [ – 19.5, – 3.7], 8; 0.025	
Monthly headache days				
All	28.0 (16.0, 28.0), 40	24.0 (0, 28.0), 19	– 4.0 [ – 12.0, – 2.0], 19; 0.012	
CWI	28.0 (16.0, 28.0), 20	28.0 (0, 28.0), 11	0 [ – 14, 0], 11; 0.053	0.1 [ – 2.7, 3.3] 19, 0.778
Bariatric surgery	28.0 (14.0, 28.0), 20	22.0 (12.0, 27.0), 8	– 4.0 [ – 16.0, 3.0], 8; 0.140	
OCT global peripapillary retinal nerve fibre layer thickness (µ)				
All	123.0 (98.0, 206.0), 39	103.0 (90.0, 114.0), 19	– 5.0 [ – 56.4, 13], 19; 0.026	
CWI	118.5 (97.3, 169.8), 20	100.0 (91.0, 118.0), 11	– 3.0 [ – 49.9, 7.4], 11; 0.123	2.5 [ – 14.0, 24.0], 19; 0.659
Bariatric surgery	134.0 (106.0, 219.0), 19	103.5 (80.3, 113.0), 8	– 12.5 [ – 99.8, 14.6], 8; 0.107	
OCT optic nerve head central thickness (µ)				
All	666.0 (513.0, 781.0), 25	626.0 (454.0, 678.5), 17	– 53.0 [ – 208.8, 38.1], 12; 0.023	
CWI	641.5 (545.5, 774.0), 12	645.5 (485.3, 681.3), 10	– 9.0 [ – 102.6, 57.3], 6; 0.752	34.5 [ – 190.0, 218.0], 12; 0.218
Bariatric surgery	715.0 (469.5, 886.5), 13	600.0 (427.0, 693.0), 7	– 95.5 [ – 390.5, 20], 6; 0.028	
OCT macular volume ganglion cell layer (mm^3^)				
All	1.1 (1.1, 1.2), 27	1.1 (1.1, 1.2), 18	0 [ – 0.1, 0], 15; 0.524	
CWI	1.1 (1.1, 1.2), 14	1.2 (1.1, 1.2), 10	0 [ – 0.1, 0], 8; 0.865	0 [ – 0.1, 0.1], 15; 0.634
Bariatric surgery	1.1 (1.1, 1.2), 13	1.1 (1.0, 1.2), 8	0 [0, 0], 7; 0.395	
Visual field perimetric mean deviation (dB)				
All	– 2.3 ( – 3.7, – 1.5), 40	– 2.2 ( – 3.2, – 0.2), 19	0.4 [ – 0.4, 1.8], 19; 0.235	
CWI	– 2.4 ( – 3.4, – 1.4), 20	– 1.6 ( – 2.9, – 0.2), 11	0.4 [ – 4.5, 4.9], 11; 0.374	1.1 [ – 1.7, 3.0], 19; 0.251
Bariatric surgery	– 2.1 ( – 5.3, – 1.6), 20	– 2.9 ( – 4.1, – 0.5), 8	0.4 [ – 1, 0.8], 8; 0.107	

*CWI* Community weight management intervention, *OCT* Optical coherence tomography

Table above showing the clinical outcomes at baseline and 12 months for the whole cohort and the two arms of Community weight management intervention and bariatric surgery

^a^Wilcoxon singed ranks test

^b^Mann–Whitney U test

**Table 4:**
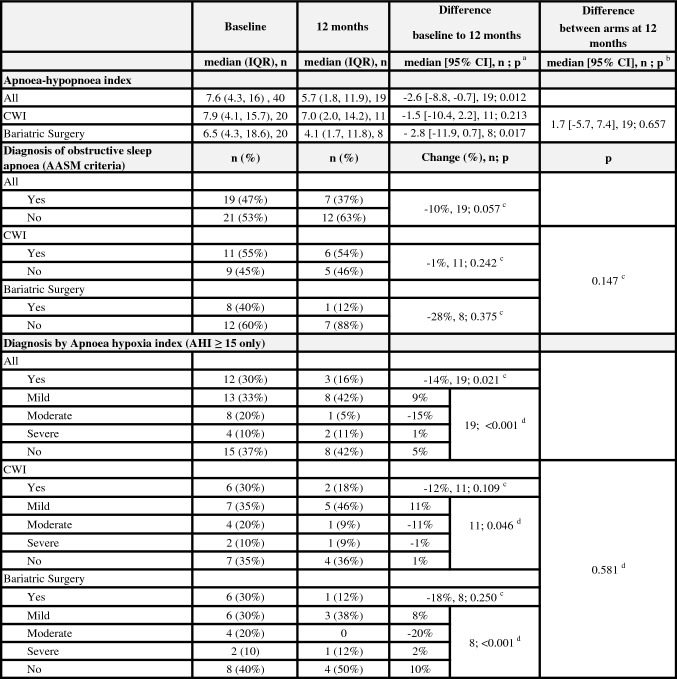
Sleep study outcomes

*AASM* American Academy of Sleep Medicine, *CWI* Community weight management intervention

Table above showing the sleep study outcomes at baseline and 12 months for the whole cohort and the two arms of community weight management intervention and bariatric surgery

^a^Wilcoxon singed ranks test

^b^Mann–Whitney *U* test

^c^Fisher’s exact test

^d^Kendall’s tau-b test

The prevalence of OSA (AASM criteria) at 12 months (compared to baseline) was 1% lower in the CWI group and 28% lower in the bariatric surgery group (Table 4). There was a significant reduction in the severity of OSA at 12 months (*p* = 0.001) with moderate OSA improving in more patients who had undergone bariatric surgery compared to the CWI (Table 4). No other additional OSA interventions were received by participants for OSA between the baseline and the 12-month clinical trial sleep studies.

### Associations of OSA on clinical outcomes in IIH

The relationship between the AHI and ICP was explored. We noted that between baseline and 12 months, improvement in the AHI correlated significantly with falling ICP (percentage change over time; *r* = 0.537, *p* = 0.018) (Fig. 2C), which became non-significant following adjusting for BMI change in the multivariable analysis (*R*^2^ 0.086, *p* = 0.348). At baseline the AHI was not significantly associated with ICP. There were no significant associations with AHI and monthly headache days in our cohort.

We found a correlation between the improving AHI and reduction in papilloedema as measured by OCT (percentage change in optic nerve head central thickness, *r* = 0.543, *p* = 0.045) (Fig. 2D). This relationship was independent of ICP when assessed in a multivariate regression model. To explore the impact of weight on the relationship of AHI and papilloedema, a multivariate regression adjusting for BMI change was also conducted. A significant association remained between AHI and papilloedema (*R*^2^ 0.522, *p* = 0.017) despite adjusting for BMI. Additionally, papilloedema was significantly associated with the oxygen desaturation index (ODI) (after adjusting for BMI (*R*^2^ = 0.504, *p* = 0.021)).

### IIH with confirmed OSA

**Fig. 3:**
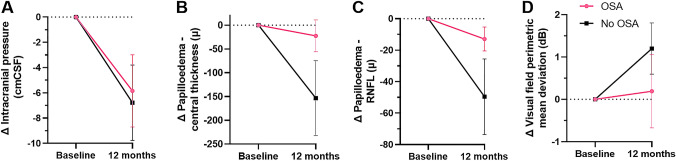
Changes according to OSA status at baseline. Mean changes (Δ) of key parameters at 12 months according to OSA status at baseline. Error bars represent standard error of the mean (SEM). OSA at baseline (*n* = 10), No OSA (*n* = 9) for A, C and D. OSA at baseline (*n* = 7), No OSA (*n* = 7) for B. *OSA* obstructive sleep apnoea, *RNFL* retinal nerve fibre layer

A descriptive observational analysis (without inference regarding statistical significance) of those with confirmed of OSA and those without was conducted. At baseline there was no significant difference between these groups in weight, ICP, papilloedema (optic nerve head volume central thickness), macular volume ganglion cell layer (a marker of optic nerve axonal loss) or visual field perimetric mean deviation (Table 5). However, amongst the patients with confirmed OSA at baseline, we observed poorer outcomes at 12 months based on the following parameters: median [95% CI] ICP, LP OP ( – 4.8 cmCSF [ – 12.3, 1.4] compared to -6.5 cmCSF [ – 13.7, 0.1]); optic nerve head volume central thickness ( – 20 µ [ – 104.4, 59.5] compared to  – 74 µ [ – 346, 39.2]); macular volume ganglion cell layer ( – 0.01 mm3 [ – 0.02, 0.02] compared to 0 mm3 [ – 0.04, 0.02]); visual field perimetric mean deviation ( – 0.3 dB [ – 2.2, 1.4] compared to 1 dB [ – 1, 2.7]) (Fig. 3A–D).

**Table 5:** Baseline characteristics according to OSA status

	OSA at baseline median (IQR), *n*	No OSA at baseline median (IQR), *n*	*p*^a^
Weight (kg)	116.3 (104.6, 143.8), 19	111.3 (101.7, 123.8), 21	0.205
Intracranial pressure (cmCSF)	35.0 (30, 39), 19	33.0 (27.8, 39.5), 21	0.361
OCT optic nerve head central thickness (µ)	615.5 (518.8, 852), 12	617 (503, 784), 15	1
OCT Macular volume ganglion cell layer (mm^3^)	1.1 (1.1, 1.2), 13	1.2 (1.1, 1.2), 14	0.105
Visual field perimetric mean deviation (dB)	− 2.4 (− 3.8, 1.6), 19	− 2.2 (− 3.6, − 1.3), 21	0.289
OCT global peripapillary retinal nerve fibre layer thickness (µ)	117 (90, 172), 21	140 (108, 215.8), 20	0.380

*OSA* Obstructive sleep apnoea, *OCT* Optical coherence tomography

Key clinical characteristics for IIH patients that underwent a sleep study based on OSA diagnosis. *P*-value represents comparison between the 2 groups of patients

^a^Mann–Whitney *U* test

Those with IIH and OSA at baseline in whom the OSA diagnosis had resolved by 12 months were observed in comparison to those in whom the OSA remained at 12 months. Amongst those in whom OSA resolved, we noted a greater reduction in ICP compared to those who still had OSA at 12 months (median [95% CI] ICP, LP OP -10.8 cmCSF [ – 22.6,  – 1.1] compared to − 3.5 cmCSF [ – 6, 3.4]. papilloedema resolution was greatest in those with the resolved OSA (median [95% CI] optic nerve head volume central thickness  – 43 µ [ – 144.8, 86.5] compared to  – 9 µ [ – 35,  – 9]), although there was no difference in visual fields outcomes.

## Discussion

A co-morbid relationship between IIH and OSA is well described but how commonly this occurs and the relevance to IIH is uncertain. In our study, the prevalence of OSA in IIH in patients with BMI ≥ 35 kg/m^2^ is high, with nearly half of them fulfilling the diagnostic criteria. Our data also suggests that treating OSA in patients with IIH may improve papilloedema. In clinic the most sensitive screening tool to identify OSA risk in IIH was the STOP-BANG questionnaire. We further noted that bariatric surgery is effective at treating OSA in IIH.

The high prevalence of OSA in IIH is greater than previously reported in the historical literature. OSA in IIH patients has been previously diagnosed through full polysomnography in 33% in a prospective study (*n* = 24, BMI 27.3–45.9 kg/m^2^) and 43% in a retrospective case-note review (*n* = 14, BMI 33.0–63.2 kg/m^2^) [12, 15]. Both studies included males and females and diagnosis of OSA was made when the AHI was ≥ 5. The higher prevalence in our cohort may reflect the larger cohort size but also trends for increasing BMI amongst IIH patients in line with the global obesity epidemic [5]. Increasing BMI is a risk factor for developing OSA [30]. Our cohort was exclusively women. Amongst men with intracranial hypertension, 30% have been reported to have sleep apnoea (case-note review, *n* = 18) [13]. The incidence of any degree of OSA in the general population is reported as 9% to 38% and is higher with increasing age, BMI and male gender [30]. Additional studies however, report OSA prevalence in the range of 7–56% in obese females [31, 32]. It is therefore likely that the prevalence of OSA observed in our IIH female cohort with obesity is akin to that expected in a general population matched cohort.

Diagnosing OSA in IIH is important as OSA is known to be independently associated with increased morbidity from Type 2 diabetes, cardiovascular disease, hypertension and increased mortality [20]. It is important to note that patients with IIH have also been previously found to have a two-fold increase in cardiovascular disease independent of the risk conferred due to co-morbid obesity [5]. The potential role of OSA in driving cardiovascular disease in IIH patients is not known. OSA also reduces quality of life, an area already identified as adversely impacted in IIH [5, 11, 33]. Hence identifying and managing OSA in IIH is likely to improve long term health outcomes in patients with IIH.

The impact of OSA on the clinical course of IIH has not been previously determined. Previous cross-sectional studies have not shown an association between OSA and IIH clinical features [14, 34]. Our results provide initial evidence that OSA in IIH impacts papilloedema. Over a 12-month study duration, our data suggests that improving OSA was associated with improving papilloedema independent of changes in BMI. Interestingly, amongst the IIH patients diagnosed with OSA, our descriptive data suggested that papilloedema and visual field recovery at 12 months was worse compared to those without OSA, despite similar changes in ICP. These data indicate that OSA may exacerbate papilloedema and visual dysfunction in addition to risks driven by ICP and weight. OSA is known to cause intermittent nocturnal hypoxia, which has been shown to exacerbate microscopic angiopathies such as diabetic retinopathy [35]. OSA has also been shown to exacerbate optic nerve ischemia in glaucoma and non-arteritic ischaemic optic neuropathy [36]. In the setting of IIH, co-existing OSA with ensuing intermittent nocturnal hypoxia could exacerbate ischemia in the papilloedema and contribute to poorer visual outcomes. We speculate that treating OSA in IIH could independently benefit optic nerve swelling and function.

We did not notice a relationship between headache measures and OSA diagnosis in this IIH cohort, although headache is an OSA symptom that is shared with IIH [11, 37]. There were no associations with the baseline AHI and monthly headache days nor with the changes at 12 months.

Using polysomnograpy is costly and resource intensive. Hence in women with IIH we found that STOP-BANG had high sensitivity (84%) and better than the Berlin and ESS questionnaires. This is consistent with a recent meta-analysis (not in IIH) that also showed that STOP-BANG was the most sensitive screening tool [38]. The ESS demonstrated the highest specificity of 74% even with our sensitivity analysis (56%). Sensitivity analysis was performed as the AASM definition and diagnosis of OSA includes excessive daytime sleepiness which was assessed via the ESS that was also used as a screening questionnaire [29]. The importance of an OSA diagnosis in IIH is thus clinically relevant as OSA should be managed, independently of the fact the patient has IIH, but treatment may also confer additional advantages to the IIH disease course. Bariatric surgery improved OSA outcomes in our IIH cohort. The significant improvement of AHI with bariatric surgery and its superiority to CWI is in line with large clinical trials and meta-analyses proposing that bariatric surgery is a definite treatment for OSA in many other patient cohorts [17]. We have also demonstrated the clinical and cost effectiveness of bariatric surgery for treating IIH [19, 39, 40].

We noted a positive association between the change in ICP and resolving OSA over the course of the study, however this was likely driven by the change in BMI. Our exploratory analysis also revealed similar improvement in ICP in patients with and without OSA. This points towards chronic changes in ICP not being directly associated with OSA in this study. However, an acute dynamic relationship between ICP and OSA has been demonstrated using concurrent ICP monitoring and sleep apnoea assessment [41]. Additionally, it has been noted that hypoxia and hypercapnia can cause cerebral vasodilation and increased cerebral blood flow with resulting elevation of ICP [41]. Furthermore in hypoxia, brain oedema in OSA may precipitate raised ICP through the apnoeic stress response and release of excitatory neurotransmitters or disruption of the blood brain barrier in susceptible individuals [42–44]. OSA may further contribute to elevated ICP by large swings and elevations in intrathorasic pressure [41, 45] with a corresponding increase venous pressure which reduces drainage at the arachnoid granulations [46]. Jugular venous pressure can be also elevated in patients with OSA resulting from neck adiposity narrowing upper airways, mouth breathing or forward head posture [47].

The main limitation of this study was that the accuracy of the home-based OSA testing could have been optimized if done with full polysomnography in a hospital setting. However, the approach used in this study reflected widespread pragmatic clinical practise. Additionally testing occurred over two nights to enable a more complete evaluation. Our results are only applicable to women with IIH and a BMI ≥ 35, and extrapolating of the results outside this cohort is not clear. While we cannot draw definite conclusions from our descriptive analysis of those with and without OSA as the study was not designed to do so, this exploratory data suggests this is worthy of study in a dedicated trial in the future.

This was the largest prospective study assessing OSA status in a cohort of IIH patients. We demonstrated that OSA is very common in women with IIH. The results suggest that OSA might be associated with worse IIH outcomes and improvements in OSA severity were associated with improvements in papilloedema. Clinicians treating women with IIH need to have low threshold to suspect OSA and the STOP-BANG questionnaire can aid screening. Whether OSA treatment can reduce the burden of IIH needs to be examined.

## Supplementary Information

Below is the link to the electronic supplementary material.

Supplementary file1 (DOCX 41 KB)

## Data Availability

Reasonable requests will provide data beginning 12 months and ending 3 years after publication of this article to researchers whose proposed use of the data is approved by the original study investigators. Proposals should be made to the corresponding author and requesters will need to sign a data access agreement.
